# Gliclazide reduced oxidative stress, inflammation, and bone loss in an experimental periodontal disease model

**DOI:** 10.1590/1678-7757-2018-0211

**Published:** 2019-02-21

**Authors:** Aurigena Antunes de ARAÚJO, Helicarlos Batista de MORAIS, Caroline Adisson Carvalho Xavier de MEDEIROS, Gerly Anne de Castro BRITO, Paulo Marcos Matta GUEDES, Sarah HIYARI, Flávia Q. PIRIH, Raimundo Fernandes de ARAÚJO

**Affiliations:** 1 Universidade Federal do Rio Grande do Norte, Programa de Pós-Graduação em Ciências Farmacêuticas, Programa de Pós-Graduação em Saúde Pública, Departamento de Biofísica e Farmacologia, Natal, Rio Grande do Norte, Brasil.; 2 Universidade Federal do Rio Grande do Norte, Programa de Pós-Graduação em Saúde Pública, Natal, Rio Grande do Norte, Brasil.; 3 Universidade Federal do Rio Grande do Norte, Programa de Pós-Graduação RENORBIO, Programa de Pós-Graduação em Biologia, Departamento de Biofísica e Farmacologia, Natal, Rio Grande do Norte, Brasil.; 4 Universidade Federal do Ceará, Programa de Pós-Graduação em Farmacologia, Programa de Pós-Graduação em Morfologia, Departamento de Morfologia, Fortaleza, Ceará, Brasil.; 5 Universidade Federal do Rio Grande do Norte, Programa de Pós-Graduação em Biologia Parasitária, Departamento de Microbiologia e Parasitologia, Natal, Rio Grande do Norte, Brasil.; 6 University of California, School of Dentistry, Section of Periodontics, Los Angeles, California, United States of America.; 7 Universidade Federal do Rio Grande do Norte, Departamento de Morfologia, Porgrama de Pós-Graduação em Biologia Funcional e Estrutural, Programa de Pós-Graduação em Ciências da Saúde, Natal, Rio Grande do Norte, Brasil.

**Keywords:** Periodontitis, Inflammation, Bone, Micro-computed tomography, Cytokines

## Abstract

**Objective:**

The aim of this study was to evaluate the effects of gliclazide on oxidative stress, inflammation, and bone loss in an experimental periodontal disease model.

**Material and Methods:**

Male albino Wistar rats were divided into no ligature, ligature, and ligature with 1, 5, and 10 mg/kg gliclazide groups. Maxillae were fixed and scanned using micro-computed tomography to quantify linear and bone volume/tissue volume (BV/TV) and volumetric bone loss. Histopathological, immunohistochemical and immunofluorescence analyses were conducted to examine matrix metalloproteinase-2 (MMP-2), cyclooxygenase 2 (COX-2), cathepsin K, members of the receptor activator of the nuclear factor kappa-Β ligand (RANKL), receptor activator of nuclear factor kappa-Β (RANK), osteoprotegerin (OPG) pathway, macrophage migration inhibitory factor (MIF), superoxide dismutase-1 (SOD-1), glutathione peroxidase-1 (GPx-1), NFKB p 50 (Cytoplasm), NFKB p50 NLS (nuclear localization signal), PI3 kinase and AKT staining. Myeloperoxidase activity, malondialdehyde and glutathione levels, while interleukin-1 beta (IL-1β) and tumor necrosis factor-alpha (TNF-α) levels were evaluated by spectroscopic ultraviolet-visible analysis. A quantitative reverse transcription polymerase chain reaction was used to quantify the gene expression of the nuclear factor kappa B p50 subunit (NF-κB p50), phosphoinositide 3-kinase (PI3k), protein kinase B (AKT), and F4/80.

**Results:**

Micro-computed tomography showed that the 1 mg/kg gliclazide treatment reduced linear bone loss compared to the ligature, 5 mg/kg gliclazide, and 10 mg/kg gliclazide treatments. All concentrations of gliclazide increased bone volume/tissue volume (BV/TV) compared to the ligature group. Treatment with 1 mg/kg gliclazide reduced myeloperoxidase activity, malondialdehyde, IL-1β, and TNF-α levels (p≤0.05), and resulted in weak staining for COX-2, cathepsin k, MMP-2, RANK, RANKL, SOD-1, GPx-1,MIF and PI3k. In addition, down-regulation of NF-κB p50, PI3k, AKT, and F4/80 were observed, and OPG staining was strong after the 1 mg/kg gliclazide treatment.

**Conclusions:**

This treatment decreased neutrophil and macrophage migration, decreased the inflammatory response, and decreased bone loss in rats with ligature-induced periodontitis.

## Introduction

Periodontitis is a chronic oral infectious disease that results from a dysregulated host immune response towards microorganisms in the dental biofilm.^1^ ^,^ ^2^ The relationship between periodontitis and other pathological conditions could be established by the immunogenic potential of host and/or bacterial products that reach the bloodstream and target distant organs and systems. Periodontal disease (PD)^3^ is a known risk factor for diabetes mellitus (DM), and there is evidence that treatment of PD reduces the incidence of inflammation and DM complications.^4^ In turn, DM is a risk factor for periodontitis. A bidirectional relationship exists between glycemic control and PD severity.^5^ The most common types of diabetes observed in primary care practice are type 1 and type 2. Type 1 diabetes is an autoimmune disease characterized by total destruction of pancreatic beta cells, whereas insulin resistance, impaired insulin secretion, and inappropriate hepatic glucose secretion characterize type 2 diabetes.^6^ The use of anti-diabetic drugs to control type 2 DM has secondary pharmacological benefits with regard to inflammatory processes.^7^ ^,^ ^8^

Gliclazide is an anti-diabetic medication, a second-generation sulfonylurea. Sulfonylureas release insulin from pancreatic cells and act on insulin-sensitive tissues to enhance glucose uptake.^9^ ^,^ ^10^ While these agents directly stimulate insulin secretion by the β-cell, Sulfonylureas have also been shown to have anti-inflammatory effects.^11^ Insulin stimulation results in the activation of distinct pathways involved in metabolic regulation, including the phosphatidylinositol-3-kinase (PI3K) cascade.^10^ Gliclazide has a direct effect on PI3K insulin-resistant skeletal muscle to enhance insulin signalling.^10^ The PI3K signaling pathway affects the inflammatory process, contributing to increased neutrophil survival,^12^ and the osteoclast differentiation pattern.^13^ PI3K expression is higher in tissue from patients with periodontitis than in healthy gingival tissue.^14^

Periodontal bone resorption is induced by osteoclasts. Receptor activator of nuclear factor-κB ligand (RANKL), its receptor RANK, and a decoy receptor osteoprotegerin (OPG) are key molecules that regulate osteoclast differentiation, recruitment, and function.^15^ Cathepsin-K is a key protease in the degradation process of bone matrix molecules. There is a positive correlation between cathepsin-K and RANKL levels, suggesting that excess production of RANKL resulted in the formation of active osteoclasts and led to cathepsin-K production in osteoclasts in the periodontium of patients with periodontitis, thus contributing to osteoclastic bone resorption.^16^ Other important proteases involved in destructive periodontal diseases are Matrix metalloproteinases (MMPs). MMPs were traditionally thought to degrade extracellular matrix components and grouped according to their substrate specificity in collagenases, gelatinases, stromelysins, matrilysins, and membrane type. Because type I collagen represents the bulk component of periodontal extracellular matrix, special attention has been paid to MMP-2 gelatinases in periodontitis.^17^

Gliclazide also decreased the expression of inflammatory markers and endothelial dysfunction in patients with type 2 diabetes.^18^ The inhibitory effect of gliclazide on AGE-induced monocyte adhesion involves a reduction in EC adhesion molecule expression and inhibition of nuclear factor kappaB (NF-kappaB) activation.^19^

The aim of this study was to investigate the roles of gliclazide in inflammation, bone loss, and PI3K/AKT pathway activation in an experimental PD rat model.

## Materials and methods

### Animals

Fifty (50) male albino Wistar rats (3 months old, 180-220 g) were bred and housed under standard conditions (12-h light/dark cycle and 22±0.1°C) and under compliance with the guidelines approved by the Animal Ethics Committee of the Federal University of Rio Grande do Norte (UFRN), Brazil (No. 066/2014). These guidelines conform to Animal Research N3CRs guidelines for Reporting of *In Vivo* Experiments for the handling and care of animals. The rats were given *ad libitum* access to food and water for the duration of the study.

### Experimental periodontitis model

Anesthesia was induced in the rats by 10% ketamine intraperitoneal injection (80 mg/kg; Vetnil, São Paulo, SP, Brazil) and 2% xylazine (10 mg/kg; Calmium, São Paulo, SP, Brazil). Experimental Periodontal Disease (PD) was induced by placement of a sterile nylon thread ligature (3-0 polysuture; Dentalcremer LTDA, São Paulo, SP, Brazil) around the crown and adjacent to the gingival tissue of the maxillary left second molar (L groups). The counterlateral side with no ligature served as the control group (no treatment, no periodontitis induction-NL group).

### Control and treatment groups

Stock solution of gliclazide (GLI) was obtained by dissolving 30 mg gliclazide (Servier, Rio de Janeiro, RJ, Brazil) in distilled water. Distilled water served as the vehicle in the NL and L groups. GLI or vehicle was administered by oral gavage (1 mL *per* rat) 1 h before ligature placement (induction of experimental PD), and once daily thereafter for 10 days. The animals were assigned randomly to the following five groups (*n*=10 each): NL, L, L with 1 mg/kg GLI, L with 5 mg/kg GLI, and L with 10 mg/kg GLI. Drug doses were selected based on those used in humans and in *in vivo* studies examining the effect of gliclazide in rats.^20^ The animals were euthanized 11 days after initial treatment with an injection of 80 mg/kg thiopental (0.5 g Thiopentax; Cristália, São Paulo, SP, Brazil). The maxillae were fixed in 10% buffered formalin for histopathological, immunohistochemical (IHC), and immunofluorescent morphological analyses. Rat maxillae were fixed in 10% buffered formalin for 24 h and stored in 70% alcohol for micro-computed tomography (micro-CT) analysis. Gingival tissues were frozen at -80°C for myeloperoxidase, malondialdehyde, glutathione, cytokine, and quantitative reverse transcription polymerase chain reaction (qRT-PCR) analyses.

### Biochemical analyses

After euthanasia, blood samples were collected by heart puncture for subsequent biochemical analysis.

Serum was obtained for biochemical analyses by centrifuging total blood without anticoagulants at 2,500 rpm for 15 min. Glucose and glycated hemoglobin (HbA1c) serum levels were determined by using standardised diagnostic kits (LABTEST^®^, São Paulo, SP, Brazil) and spectrophotometry (BIO2000 BIOPLUS, São Paulo, SP, Brazil).

### Micro-CT analysis

Rat maxillae were scanned in a micro-CT device (model 1172; SkyScan, Kontich, Belgium). The micro-CT files were converted to Digital Imaging and Communications in Medicine format and imported into the Dolphin^®^ software package (Dolphin Imaging, Chatsworth, CA, USA) for linear bone loss analysis. The maxillae were oriented with the second molar, cement enamel was identified in the axial plane, and linear bone distances on the sagittal plane were recorded for the second mesial molar from the CEJ to the alveolar bone crest (ABC). Two additional mesial second molar palatal measurements were taken 0.3 mm from the middle of the crown.

Bone volume/tissue volume (BV/TV) samples were oriented using the DataViewer software package (ver. 1.5.2; Bruker, Billerica, MA, USA) for volumetric analysis. The maxillae were oriented with the second molar CEJs parallel to each other in the sagittal and coronal planes. The crowns of the first, second, and third molars were visible in the axial plane. After orientation, the files were imported into CTAn (ver. 1.16; Bruker) for volumetric analysis. A 40-slice volume set at a threshold of 75 was considered to be the region of interest for analysis. Analysis started at the slice in which furcation first appeared, proceeding apically. BV/TV percentage values were recorded and averaged for each group (*n*≥4/group for all micro-CT analyses).

### Histopathological analysis

Alveolar bone specimens were harvested, fixed in 10% neutral-buffered formalin (24 h), and demineralized in 5% nitric acid for 14 days. Subsequently, the specimens were dehydrated, embedded in paraffin, sectioned along the molars in the coronal plane, and stained with hematoxylin and eosin. Sections (4 m) were analyzed by light microscopy (40× magnification) in the Department of Morphology at UFRN.

A histologist scored inflammatory cell influx and integrity of the alveolar bone and cementum in a single-blind manner. Scores were assigned as follows: 0, no or sparse inflammatory cell infiltration, restricted to the marginal gingiva, with preservation of the alveolar process and cementum; 1, moderate inflammatory cell infiltration of the entire gingival insert with minor alveolar resorption and intact cementum; 2, marked inflammatory cell infiltration of the gingiva and periodontal ligament with marked degradation of the alveolar process and partial destruction of the cementum; and 3, marked inflammatory cellular infiltration with complete resorption of the alveolar process and severe destruction of the cementum.^21^ The Kruskal-Wallis test followed by Dunn’s test were used to compare medians (GraphPad Prism 5.0 software, La Jolla, CA, USA). A p-value≤0.05 was considered representative of statistical significance.

### Immunohistochemical analysis

Maxillae tissue was deparaffinized and rehydrated. Gingival and periodontal tissue slices (4 μm) were washed with 0.3% Triton X-100 in phosphate buffer, quenched with endogenous peroxidase (3% hydrogen peroxide), and incubated overnight at 4°C with primary antibodies against the following proteins (all antibodies 1:400): receptor activator of the NF-κB ligand (RANKL), superoxide dismutase-1 (SOD-1), receptor activator of NF-κB (RANK), glutathione peroxidase-1 (GPx-1), osteoprotegerin (OPG), matrix metalloproteinase 2 (MMP-2), cathepsin K, and cyclooxygenase-2 (Santa Cruz Biotechnology, INTERPRISE, São Paulo, SP, Brazil) and incubated for 30 min with a streptavidin/*horseradish peroxidase*-conjugated secondary antibody (Biocare Medical, Concord, CA, USA). Immunostaining was visualized using colorimetric detection (Biocare Medical, Dakota, USA). The staining status was identified as either negative or positive; positive staining was defined as the presence of brown chromogen. Staining intensity and the proportion of immunopositive cells were examined independently by two pathologists by light microscopy and recorded. Intensity of staining (IS) was graded on a 0 to 2 scale according to the following semi-quantitative assessment: 0 = no detectable staining, 1 = weak staining; 2 = strong staining.^22^

### Immunofluorescence

Three periodontal tissue sections from each animal (*n*=3/group) were deparaffinized in xylene and washed in a series of concentrations of ethanol and Phosphate-Buffered Saline (PBS) buffer. Antigen retrieval was performed by placing the sections in 10 mM sodium citrate solution with 0.05% Tween 20 for 40 min at 95°C. The sections were incubated overnight at 4°C with rabbit anti-macrophage migration inhibitory factor (MIF) primary antibody (1:100 in 1% normal goat serum; Santa Cruz Biotechnology, Miami, USA), NFKB P50 (cytoplasm), NFKB p50 NLS (Nuclear localization signal), PI3K and AKT. The samples were incubated with Alexa Fluor 488-conjugated goat anti-rabbit secondary antibodies (1:500 in 1% bovine serum albumin) and counterstained with DAPI (Sigma, Miami, USA)*.* Fluorescent images were then obtained (laser scanning microscope 710, 20X objective; Carl Zeiss, Jena, Germany). Tissue reactivity using laser scanning was assessed by computerized densitometry analysis of the digital images.

### Myeloperoxidase assay

The extent of neutrophil accumulation in gingival tissue samples was measured by assessing myeloperoxidase (MPO) activity. Four samples of gingival tissue *per* group were harvested as described above and stored at -80°C until required for the assay. Gingival tissue was homogenized in hexadecyltrimethylammonium bromide (1:20 wt:vol), subjected to two freeze/thaw cycles, and centrifuged at 5000 rpm for 20 min. MPO activity (in units of MPO/milligram of tissue) was determined at 450 nm using a previously described colorimetric method.^23^

### Malondialdehyde levels

Malondialdehyde (MDA) levels in gingival tissues were measured using a previously described assay.^24^ Gingival samples were suspended in 1:5 (wt:vol) Tris hydrogen chloride (HCl) buffer and minced with scissors for 15 s on an ice-cold plate. The resulting suspension was homogenized for 2 min with an automatic Potter homogenizer and vortexed, and 1-methyl-2-phenylindole and 37% HCl were added. The vortexed solution was then incubated at 45°C for 40 min. Solutions were then cooled on ice and centrifuged for 10 min at 15,000 *g*. Sample absorbance was measured at 586 nm (as nanomoles/gram of tissue).

### Non-protein *sulfhydryls* assay

Glutathione (GSH) levels in gingival tissues were measured as a marker of antioxidant activity.^25^ Five gingival samples were harvested *per* group and stored at -70°C until required for the assay. Gingival tissue homogenate (0.25 mL 5% tissue solution prepared in 0.02 M Ethylenediamine tetraacetic acid/EDTA) was added to 320 µL distilled water and 80 µL 50% *Trichloroacetic acid/*TCA. Samples were centrifuged at 3000 rpm and 4°C for 15 min. Then, 400 µL supernatant was added to 800 µL 0.4 M Tris buffer (pH 8.9) and 20 µL 0.01 M 5,5’-dithio-bis-[2-nitrobenzoic acid]/DTNB. Sample absorbance was measured at 420 nm. The results were reported as units of GSH *per* milligram of tissue.

### Interleukin-1β and tumor necrosis factor-α assay

Gingival tissue samples were stored at -70°C until utilized for the assay.^26^ Gingival samples (five/group) were used to determine levels of interleukin-1β (IL-1β) and tumor necrosis factor-α (TNF-α) using an enzyme-linked immunosorbent assay kit (R&D Systems, Minneapolis, MN, *USA*) according to the manufacturer’s guidelines, as previously described.^27^ All samples were within the wavelength range of ultraviolet-visible spectrophotometry (absorbance measured at 490 nm).

### RNA extraction and qRT-PCR

Total RNA from gingival tissues was extracted using the Trizol^®^ reagent (Life Technologies, Los Angeles, USA) and chloroform (VETEC, São Paulo, SP, Brazil), as previously described.^28^ RNA was isolated and purified using the SV Total RNA Isolation System (Promega Corporation, Madison, WI*,* USA). The RNA concentration was determined from the optical density at a wavelength of 260 nm (using a unit equivalent to 40 μg/mL RNA). After isolation, 0.4 μg total RNA was reverse transcribed to cDNA (High-Capacity cDNA reverse transcription kit, Thermofisher, Los Angeles, USA) in a total volume of 20 μL. The reaction mixture was incubated at 42°C for 60 min. The reaction was terminated by heating at 70°C for 10 min. The cDNA was stored at -80°C until further use.

Gene expression was evaluated by PCR amplification using primer pairs based on published sequences from *Rattus norvegicus*. The PCR primer sequences were as follows: GAPDH forward AACTTGGCAT CGTGGAAGG, reverse GTGGATGCAG GGATGATGTT C; PI3K forward CAGGAAAGCA GGAAAAGTGC, reverse CGAAGACCAG CTCAATA; AKT forward TCACCTCTGA GACCGACACC, reverse ACTGGCTGAG TAGGAGAACT G; NF-κB from v-rel avian reticuloendotheliosis viral oncogene homolog A (Rela) mRNA forward TCTGCTTCCA GGTGACAGTG, reverse ATCTTGAGCT CGGCAGTGTT; and F4/80 forward GCCCTTCCAACTCATCATGT, reverse AGGGAATCCTTTTGCATGTG.

qRT-PCR was performed using fast SYBR green master mix and a Step One Plus thermocycler (both from Applied Biosystems, Foster City, CA, USA) according to the manufacturer’s instructions. cDNA (2.0 μL of each sample) was added to the 1X PCR master mix to make a final volume of 20 μL. PCR conditions were as follows: 95°C for 5 min and 40 cycles of 30 s at 95°C, 30 s at 52–60°C (based on the target), and 60 s at 72°C. The quantitative fold change relative to the NL group was calculated using the comparative ΔΔCt method, where Ct is the cycle number at which fluorescence first exceeds the threshold. Ct values were obtained for each sample by subtracting values for GADPH Ct from the target gene Ct value. The specificity of resulting PCR products was confirmed by melting curve analysis.

### Statistical analysis

**Figure 1:**
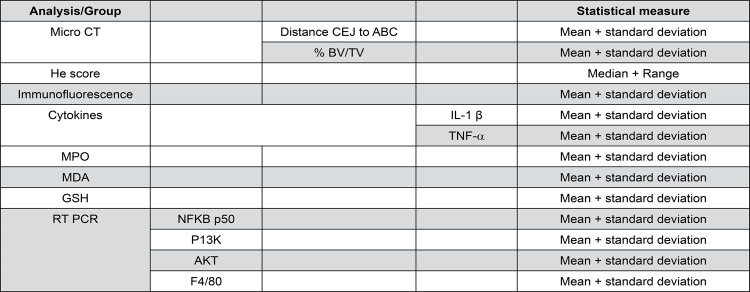
Analysis/group and statistical measure (central tendency and variability)

Data from the linear and volumetric bone loss analyses were represented as averaged values for each group±mean standard errors (Figure 1). Analysis of variance followed by Bonferroni’s test was used to compare the groups. The Kruskal-Wallis test followed by Dunn’s test were used to compare medians (GraphPad Prism 5.0 software, La Jolla, CA, USA). A p-value≤0.05 was considered representative of statistical significance.

## Results

### Biochemical analyses

Analyses were performed to determine the effect of GLI on systemic changes. Levels of glucose showed increased in L group (176.8 mg/dl) compared with NL (115.7 mg/dl). The rats with experimental periodontal disease showed low levels of glucose compared to the L groups: 1 mg/kg GLI (137.2 mg/dl), 5 mg/kg GLI (144.1 mg/dl) and 10 mg/kg GLI (135.7 mg/dl) (Table 1).

**Table 1:** Determination of the glucose and HbA1c. Biochemistry of periodontal disease experimental model

Groups	Glucose (mg/dl)	HbA1c (%)
NL	115.7±18.86	3.5±0.1
L	178.8±83.58	3.8±0.3
GLI 1 mg/kg	137.2±34.37	3.5±0.4
Gli5 5 mg/kg	144.1±22.5	3.3±0.2
Gli 10 mg/kg	135.7±40.50	2.6±0.09*

*p<0.05, (L compared among NL and GLI treatments)

Glycated hemoglobin (HbA1c) presented percentages within the range of values considered normal of 3.85%+0.92%,^29^ with values of NL (3.5%), L (3.8%), 1 mg/kg GLI (3.5%) and 5 mg/kg GLI (3.3%), respectively. The values for HbA1c in the 10 mg/kg GLI were below the reference values of 2.61%, p<0.05 (Table 1).

### Micro-computed tomography assessment of experimental PD

**Figure 2:**
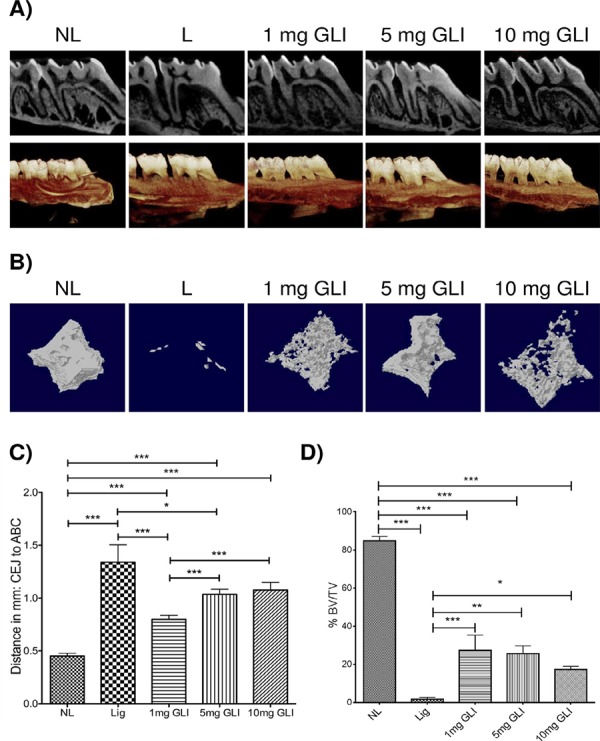
Radiographic evaluation after gliclazide (GLI) treatment and induction of experimental periodontitis. (A) Representative sagittal 2D and 3D micro-CT images from the no ligature (NL), ligature (L), and GLI 1, 5, and 10 mg/kg groups. (B) Representative sagittal 2D and 3D micro-CT images from the NL, L, and GLI 1, 5, and 10 mg/kg groups. (C) Graph representing linear bone loss in the area of the mesial second molar. Distance (in millimeters) from the cementoenamel junction to the alveolar bone crest. Data are mean±standard area of the mean. *p<0.05, *p<0.01, ***p<0.001 (n≥3 for all groups/time points). (D) Graph representing the bone volume/total volume percentage in the area below the second molar bifurcation. Data are mean±standard area of the mean. *p<0.05, *p<0.01, ***p<0.001 (n≥3 for all groups/timepoints). Analysis of variance followed by Bonferroni’s test

Specimens from all rats treated with GLI (1, 5, and 10 mg/kg) showed significantly more linear bone loss (Distance CEJ to ABC) compared to the NL group (mean 0.4515 ± standard deviation 0.026 mm; Figure 2C). The group treated with 1 mg/kg GLI (mean 0.8 ± standard deviation 0.29) showed a more dramatic reduction in linear bone loss (Distance CEJ to ABC) compared to the L group (mean 1.34±standard deviation 0.163 mm, p<0.001), and compared with other GLI-treated groups: 5 mg/kg (mean 1.04±standard deviation 0.32, p<0.001) and 10 mg/kg (mean 1.08±standard deviation 0.47, p<0.001) (Figure 2C). A similar pattern was observed volumetrically (Figure 2B). L group and the groups treated with GLI (1, 5, and 10 mg/kg) showed significantly more bone volume/tissue volume (BV/TV) loss compared to the NL group (mean 84.73±standard deviation 5.25 mm; Figure 2D). The group treated with 1 mg/kg GLI (mean 27.5±standard deviation 17.5) showed reduced bone volume/tissue volume (BV/TV) loss compared to the L group (mean 1.77±standard deviation 1.82 mm, p<0.001). The 5 mg/kg (mean 1.04±standard deviation 0.32, p<0.001) and 10 mg/kg GLI-treated groups (mean 1.08±standard deviation 0.47, p<0.001) decreased reduction in bone volume/tissue volume (BV/TV) loss compared with the L group (mean 1.77±standard deviation 1.82 mm, p<0.01 and p<0.05, respectively) (Figure 2D).

### Histopathological analysis

**Figure 3:**
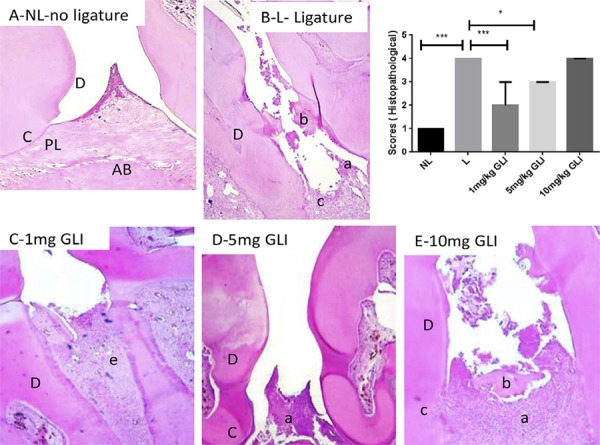
Histological analysis of maxillae from rats with periodontal disease. (A) Normal periodontium (no ligature group) showing alveolar bone integrity, absence of or only discrete cellular infiltration, and preserved alveolar bone. (B) Periodontium from a rat with periodontitis (ligature group) showing alveolar bone and cementum resorption (discontinuous cementum) and accentuated inflammatory cell infiltration. (C) Periodontium from a rat with periodontitis treated with 1 mg/kg gliclazide (GLI) showing reduced inflammation and decreased alveolar bone loss. (D) Periodontium from a rat treated with 5 mg/kg gliclazide showing moderate cellular infiltration. (E) Periodontium from a rat treated with 10 mg/kg gliclazide showing no reduced inflammation and increased experimental PD. PL, periodontal ligament; D, dentin; AB, alveolar bone; C, cementum; a, inflammatory process; b, bone loss; c, resorption of cementum; d decreased inflammation process. Sections were stained with hematoxylin and eosin. Original magnification, 40×. Histopathological scores. *p<0.05, **p<0.01, ***p<0.001

Histopathological analysis showed that structures of the periodontal tissue, marginal gingiva and ligament, cementum, and alveolar bone were preserved in the NL group (Figure 3A). The L group showed infiltration of inflammatory cells in association with destruction of the cementum and alveolar process (Figure 3B). The median histopathological score for this group was 3 (range, 3–3; Figure 3). The group treated with 10 mg/kg GLI exhibited infiltration of inflammatory cells and extensive destruction of the cementum and alveolar process, with an average histopathological score of 3 (range, 3–3; *p*>0.05; Figures 3E). Histopathology revealed slight cellular infiltration restricted to the gingival area, with preserved alveolar bone and cementum in the 1 mg/kg GLI and 5 mg/kg GLI groups. Average scores in these groups were 1 (range, 1–1; *p*<0.01) and 3 (range, 2–3; *p*<0.05), respectively (Figures 3C and D).

### Immunohistochemical analysis

**Figure 4:**
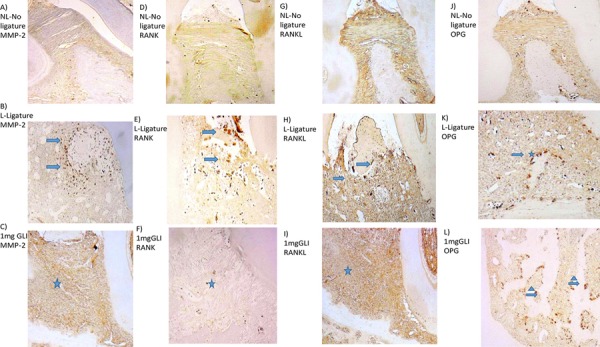
Photomicrographs of periodontal tissue from rats with periodontal disease treated with 1 mg/kg gliclazide (GLI) and control groups (no ligature and ligature), showing immunoreactivity to MMP-2, RANK, RANKL, and OPG. (A, D, G, J) Rats with no ligature. (B, E, H, K) Rats with ligature. (C, F, I, L) Rats with ligature treated with 1 mg/kg GLI. Arrow indicates high or moderate labeling in inflammatory cells in periodontal ligament or alveolar bone. Asterisk and arrow indicates mild labeling in the periodontal ligament. Triangle and arrow indicate high labeling of osteoclasts. Asterisk indicates mild labeling in the periodontal ligament or alveolar bone. Images are shown at 40× magnification

**Figure 5:**
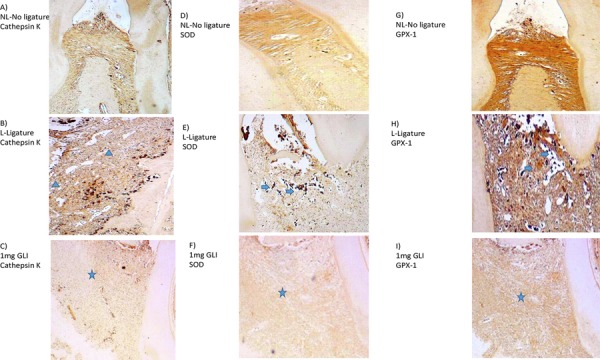
Photomicrographs of maxillary periodontal tissue from rats with periodontal disease treated with 1 mg/kg gliclazide (GLI) and control groups (no ligature and ligature), showing immunoreactivity to cathepsin (CAT), superoxide dismutase-1 (SOD-1) and glutathione peroxidase-1 (GPx-1). (A, D, G) Rats with no ligature. (B, E, H) Rats with ligature. (C, F, I) Rats with ligature treated with 1 mg/kg GLI. Arrow indicates high or moderate labeling in the periodontal ligament or alveolar bone. Asterisk indicates mild labeling in the periodontal ligament or alveolar bone. Triangle indicates intense labeling in alveolar bone. Images are shown at 40× magnification

Periodontal tissue from the L group showed a marked strong in immunostaining for MMP-2, RANK, RANKL, cathepsin K, SOD-1, and GPx-1 compared with that from the NL group (Figures 4, 5). Samples from animals treated with 1 mg/kg GLI showed weak immunostaining for MMP-2, RANKL, RANK (Figure 4C, F, and I, respectively), cathepsin K, SOD-1, and GPx-1 (Figure 5C, F, and I, respectively), and strong staining for OPG (Figure 4L).

### Immunofluorescence

**Figure 6:**
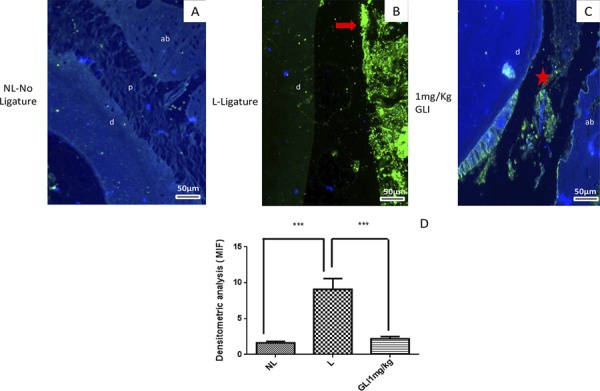
Gliclazide (GLI) modulates macrophage migration inhibitory factor (MIF) expression. Representative confocal photomicrographs of MIF immunoreactivity in periodontal specimens from each group (green) with DAPI nuclear counterstaining (blue). (A) Sample from the no ligature group showing discrete MIF labeling in dentin (d), periodontium (p), or alveolar bone (ab); (B) Sample from the ligature group shows strong labeling (red arrows) in the periodontal area. (C) Sample from the 1 mg/kg GLI group showing diffuse, weak MIF labeling (red star). Scale bar 50 µm, 200× magnification. (D) Densitometry analysis confirmed significantly decreased MIF immunoreactivity in the 1 mg/kg GLI group, which was blocked in the ligature group. Five immunofluorescence sections from each animal (n=3/group) were analyzed. ***p<0.001, Kruskal–Wallis test followed by Dunn’s test

MIF immunoreactivity was indicated by strong labeling in the periodontal area of samples from the L group (Figure 6B). Discrete MIF labeling was observed in dentin, periodontium, or alveolar bone in samples from the NL group (Figure 6A), and weak, diffuse labeling was observed in samples from the 1 mg/kg GLI group (Figure 6C). Densitometry analysis confirmed significantly decreased MIF immunoreactivity in the 1 mg/kg GLI group relative to the L group (*p*<0.001; Figure 6D).

**Figure 7:**
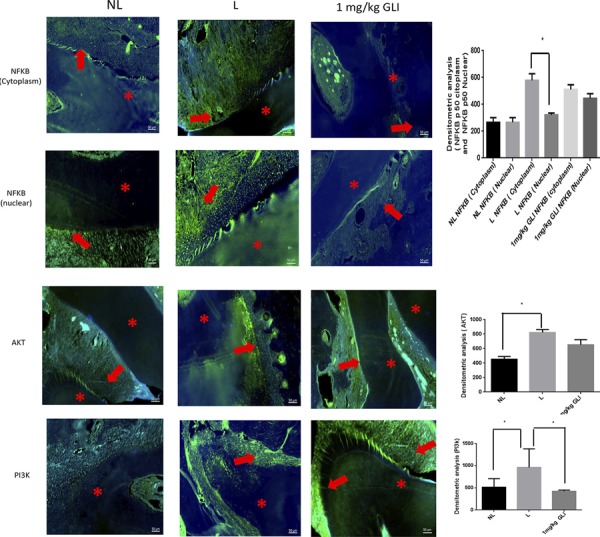
Gliclazide (GLI) modulates NFKB P50 (Cytoplasm), NFKB P50 (Nuclear), AKT, PI3K immunoreactivity in periodontal specimens from each group (green) with DAPI nuclear counterstaining (blue). Densitometry analysis confirmed significantly increased NFKB p50 (cytoplasm) immunoreactivity relative NFKB p50 (Nuclear) in the L group (p<0.05). Representative confocal photomicrographs of PI3K immunoreactivity was indicated by strong labeling in the periodontal area of samples from the L group. Discrete Pi3K labeling was observed in alveolar bone in samples from the NL group and weak, diffuse labeling was observed in samples from the 1 mg/kg GLI group, p<0.05. Asterisks: Dental tissue; Arrows (red): Cells. Scale bar 50 µm, 200× magnification. Five immunofluorescence sections from each animal (n=3/group) were analyzed. ***p<0.001, Kruskal-Wallis test followed by Dunn’s test

Densitometry analysis confirmed significantly increased NFKB p50 (cytoplasm) immunoreactivity relative NFKB p50 (Nuclear) in the L group (*p*<0.05; Figure 7). PI3K immunoreactivity was indicated by strong labeling in the periodontal area of samples from the L group (Figure 7). Discrete PI3K labeling was observed in dentin, in samples from the NL group (Figure 7), and weak, diffuse labeling was observed in samples from the 1 mg/kg GLI group (p<0.05; Figure 7).

### Oxidative stress, inflammation, and gene expression analysis

**Figure 8:**
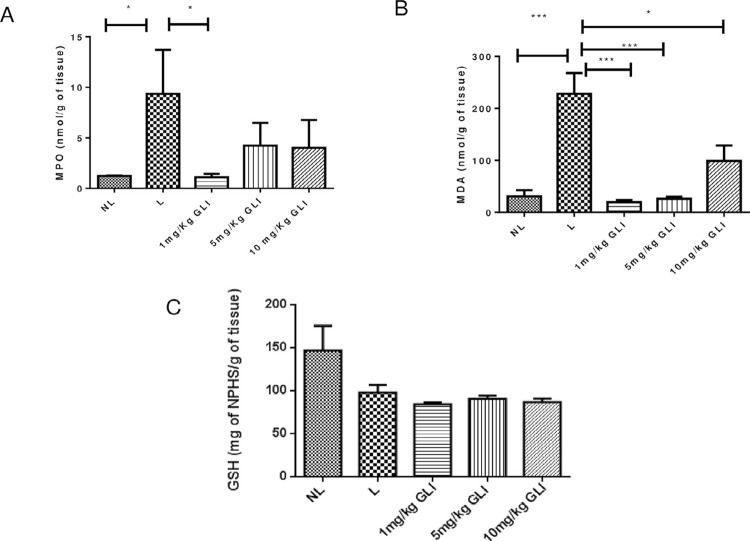
Levels of (A) MPO, (B) MDA, and (C) GSH in gingival tissue in the no ligature, ligature, and 1, 5, and 10 mg/kg gliclazide groups. *p<0.05, **p<0.01

**Figure 9:**
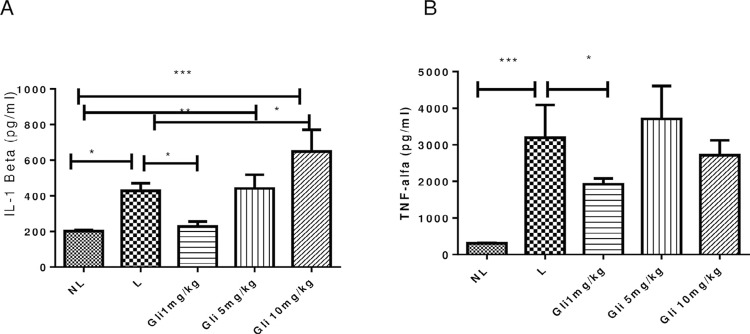
Levels of (A) IL-1β and (B) TNF-α in gingival tissue in the no ligature, ligature, and 1, 5, and 10 mg/kg gliclazide groups. *p<0.05, **p<0.01

**Figure 10:**
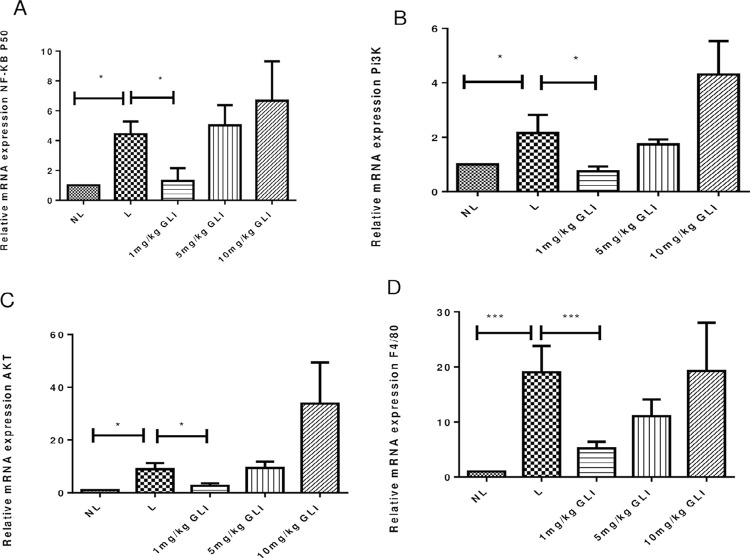
Effects of gliclazide (GLI) on (A) NF-κB p50, (B) PI3K, (C) AKT, and (D) F4/80 mRNA expression in gingival tissue from rats without periodontal disease (no ligature) and those with periodontal disease (ligature and 1 mg/kg GLI groups). The expression of NF-κB p50, PI3K, AKT, and F4/80 mRNA was decreased in the 1 mg/kg GLI (p<0.05 and p<0.001) and no ligature (p<0.001) groups. Analysis of variance followed by Bonferroni’s test (n=5 animals/group)

Analysis of gingival tissue samples showed increased MDA levels in the L group compared to the NL group (*p*<0.001). All GLI-treated groups showed reduced MDA levels compared with the L group (1 and 5 mg/kg GLI, *p*<0.001; 10 mg/kg GLI, *p*≤0.05; Figure 8A). GSH levels did not differ significantly from those in the L group (Figure 8C). GLI at a dose of 1 mg/kg prevented the increase of MPO activity (*p*≤0.05, Figure 8B) and IL-1β and TNF-α content (both *p*<0.05; Figure 9A and B) induced by ligature placement in gingival tissue, whereas the other doses analyzed showed no significant effect. Animals treated with 1 mg/kg GLI showed reduced gene expression levels of NF-κB p50, PI3K, AKT (all *p*<0.05), and F4/80 (*p*<0.001) compared with the L group (Figure 10A–D).

## Discussion

Gliclazide at a dose of 1 mg/kg led to significantly reduced oxidative stress, inflammation, and bone loss, which is in agreement with the results of other studies.^30^ ^-^ ^32^ GLI-treated rats showed decreased MDA levels compared to untreated rats.

Moreover, animals treated with 1 mg/kg gliclazide showed weak immunostaining for SOD-1 and GPx-1 in this study. According to Liu, et al.^33^ (2014), higher SOD levels in patients with periodontitis can be explained by the increased stimulation of SOD production to protect against biological superoxide generation in periodontal inflammation. Gliclazide presented antioxidant activity because it showed free radical scavenging properties. This property is due to the presence of a single aminoazabicyclooctane ring present in the sulfonylureas.^34^

Toxic reactive oxygen metabolites may also initiate and/or amplify inflammation via the up-regulation of several different genes involved in inflammatory response, including NF-κB.^35^ NF-κB is a ubiquitous transcription factor and pleiotropic regulator of numerous genes involved in immune and inflammatory responses. Activation of NF-κB amplifies the inflammatory response by up-regulating the production of pro-inflammatory cytokines.^36^ We found down-regulation of NF-κB, reduced IL-1β and TNF-α levels in the group treated with 1 mg/kg GLI. A high concentration of NFKB p50 NLS after treatment group with 1 mg/kg GLI was found in the leucocytes nucleus; however, the high concentration of NFKB p50 in the ligature group was found in leucocyte cytoplasm, indicating translocation and activity of proteins. These cytokines have been implicated in MMP and cathepsin K activation. Osteoclasts secrete cathepsin K to break down collagen, the major component of the non-mineral protein matrix of the bone.^37^

The RANKL/RANK/OPG signaling pathway is implicated in bone resorption through its key function in osteoclast differentiation and activation, as well as in inflammatory response. This central element of osteoimmunology has been suggested to be disturbed in several diseases, including periodontitis, as it is a predisposing factor for this disease.^15^ Experimental periodontitis studies have shown that cytokines like IL-1 and TNF-alpha induce osteoclastogenesis via the RANKLRANK-OPG pathway.^15^

The periodontal tissues from, 1 mg/kg GLI group showed weak immunostaining for MMP-2, RANKL and cathepsin K, as well as less tissue damage and strong OPG immunostaining. This group also showed weak immunostaining for RANKL and strong immunostaining for OPG. The tissues from 1 mg/kg and 5 mg/kg GLI groups revealed the presence of mild cellular infiltrate restricted to the gingival area with preserved alveolar bone and cementum.

Our data showed that the best results related to bone loss were found when gliclazide administration at the lower doses of 1 mg/kg translates the drug dosage from animal to human dose,^20^ as the therapeutic dose of gliclazide in humans occurs in a range of 30 mg-120 mg/day. We find that the dose of 10 mg/kg in animals is close to the maximum therapeutic dose in humans (approximately 120 mg/day, while the dose of 5 mg is equivalent to 60 mg/day and 1 mg is equivalent to 12 mg/day, below the therapeutic dose). The related biochemical data showed that animals with periodontal disease tend to have an increase in glucose levels. The fasting glycemia data may present different confounding variables; among them, the increase in glycemia can vary due to the stress during the manipulation process of the animals, which does not necessarily refer to periodontal disease or hypoglycemic effect. Data regarding glycated hemoglobin were considered more reliable data and presented a significant percentage reduction in the animals treated with gliclazide at a dose of 10 mg/kg, suggesting a hypoglycemic effect at this dose and increased bone loss in periodontal disease. Another study showed than metformin significantly activated AMPK in dose- and time-dependent manners, and induced endothelial nitric oxide synthase (eNOS) and bone morphogenetic protein-2 (BMP-2) expressions.^38^

A reduction in PI3K/AKT and NFKB p50 levels was observed in rats treated with 1 mg/kg GLI in this study. This reduced pathway activation resulted in a lower neutrophil survivability, as confirmed by the reduced MPO activity. Gliclazide seems to dysregulate the PI3K/AKT pathway, thereby reducing neutrophil survivability. Early activation of the PI3K/AKT pathway is a major step in the inhibition of apoptosis in *Anaplasma phagocytophilum*-infected neutrophils,^12^ and the role of the NFKB and PI3K pathways in neutrophil apoptosis inhibition in periodontal inflammation has been demonstrated.^14^ Treatment with 1 mg/kg GLI resulted in a more dramatic reduction in bone loss compared with the other gliclazide treatments.

The F4/80 glycoprotein is a member of the EGF-transmembrane 7 family and has been established as a specific cell-surface marker for murine macrophages.^39^ We observed reduced F4/80 gene expression in the group treated with 1 mg/kg GLI in this study, which was corroborated by the MIF immunofluorescence assay. In the context of PD, MIF has a role in controlling bacterial growth, but it more significantly contributes to the progression of bone loss by directly affecting osteoclast differentiation and activity.^13^

High doses of gliclazide (e.g., 10 mg/kg) activate the PI3K/AKT pathway and increase periodontal bone loss. Some cytokines activate the PI3K/AKT pathway, leading to osteoclastogenesis.^40^ An increase in the inflammatory cytokine IL-1β was observed in the 10 mg/kg GLI group. IL-1 has been reported to stimulate osteoclastogenesis through two parallel events: direct enhancement of RANKL expression and suppression of OPG expression.^40^

## Conclusion

We conclude that the use of GLI at a 1-mg/kg dose in rats reduces the formation of lipid peroxidation products, prevents PI3k signaling, and decreases IL-1β and TNF-α levels, which in turn reduce MMP-2, cathepsin K, and RANKL levels. Reduced gene expression of *PI3K/AKT* pathway genes was observed together with lower neutrophils infiltration, accompanied by lower neutrophil survivability and reduced migration of macrophages (MIF), which may have contributed to the reduction in linear bone loss.
